# Comparing the express and enhanced workflows for restoration of hip length and combined offset using the Mako robotic arm-assisted total hip arthroplasty

**DOI:** 10.1302/2633-1462.72.BJO-2025-0288.R1

**Published:** 2026-02-09

**Authors:** Tim Cheok, Veronica Pajnic, Julie F. Vermeir, Yvana Toh, William J. Donnelly, Anthony M. Silva

**Affiliations:** 1 Department of Orthopaedic Surgery, The Prince Charles Hospital, Brisbane, Australia; 2 College of Medicine and Public Health, Flinders University, Bedford, Australia; 3 Graduate School of Medical Sciences, University of Groningen, Groningen, Netherlands; 4 Department of Orthopaedic Surgery, St Vincent’s Private Hospital Northside, Brisbane, Australia; 5 University of Queensland Centre for Clinical Research, Faculty of Medicine, The University of Queensland, Brisbane, Australia; 6 STARS Education and Research Alliance, Surgical Treatment and Rehabilitation Service (STARS), The University of Queensland and Metro North Health, Brisbane, Australia; 7 Faculty of Medicine, The University of Queensland, Herston, Australia

**Keywords:** Robotic, Mako, Hip, Enhanced, Express, total hip arthroplasty (THA), Robotic arm, postoperative instability, retrospective cohort study, femoral components, limb length discrepancies, acetabular defects, Mann-Whitney U test, Fisher’s exact test

## Abstract

**Aims:**

The Mako system is one of the most widely used systems for robotic arm-assisted total hip arthroplasty (THA). Two workflows for femoral preparation exists for this system – the enhanced and the express workflow.

**Methods:**

We performed a retrospective cohort study comparing the accuracy of each workflow in restoring the patient’s combined offset and correcting the hip length discrepancy. The Mako derived values were compared against measured values and assessed with Bland-Altman plots and Theil’s median slope. Secondary outcomes of interest included comparison of the measured combined offset and hip length discrepancy, surgical time, incidence of postoperative instability/dislocation, as well as pin-site related complications between the two groups.

**Results:**

A total of 81 patients were identified from our database: 61 in the enhanced group and 20 in the express workflow group. Bland-Altman plots demonstrated agreement between the measurements for both hip length discrepancy and combined offset. There was no significant difference in the measurement of hip length discrepancy or combined offset difference between the two groups; however, the magnitude of the latter was better in the enhanced (median 1.50 mm) compared with the express workflow group (median 3.13 mm). There was no significant difference in measured combined offset (p = 0.254), hip length discrepancy (p = 0.425), or surgical time (p = 0.548). Lastly, there were no patients with postoperative instability/dislocation nor pin-site related complications in either group.

**Conclusion:**

Both techniques provide excellent outcomes with minimal risk of complications when performing a Mako robotic arm-assisted THA.

Cite this article: *Bone Jt Open* 2026;7(2):185–194.

## Introduction

In patients with moderate to severe osteoarthritis of the hip, total hip arthroplasty (THA) has been proven to be an effective treatment strategy, not only for providing pain relief but also for restoring function.^1^ The demand for THA is projected to increase globally,^2^ with a recent forecast predicting a 37% increase in the UK by 2060.^3^ Technical objectives in a THA include minimizing limb length discrepancies and restoration of the combined offset.^4^ Robotic arm-assisted THA has been thought to be a useful adjunct for surgeons to achieve these technical objectives.

The Mako robotic arm-assisted THA (Stryker, USA) was first introduced in 2011 and is one of the most widely used systems globally.^5^ It is a semi-active system with haptic feedback, which allows for accurate bony resection while preserving the soft-tissue envelope.^6^ Several previous studies have found that the Mako system improved acetabular component placement within the Lewinnek and Callanan safe zones.^7-9^ In terms of femoral preparation, the Mako robotic system provides two different workflows, which can be used based on surgeon preference. The enhanced workflow requires complete mapping of the proximal femur and acetabulum in comparison to the 3D plan. In the enhanced workflow, femoral resection and broaching are performed and verified with Mako assistance, allowing for adjustment of cup and femoral component placement to achieve an appropriate combined anteversion. This has previously been shown to further minimize the risk of impingement.^10^ Sugano et al^11^ have demonstrated a high degree of precision of femoral component anteversion when inserted under Mako assistance. Interestingly, in their series, the clinical accuracy of the cemented Exeter femoral components was better than the uncemented Accolade II femoral components. In contrast, the express workflow uses the robotic arm for acetabular preparation only, but allows the surgeon to assess for hip length discrepancies and the combined offset intraoperatively.^12^

A previous study by Kayani et al^13^ found that the Mako robotic arm-assisted THA preserved the patient’s native offset using the express workflow, as compared to conventional THA; however, there was no difference between the two groups in achieving planned limb length corrections. Further, a cadaveric study by Nawabi et al^14^ demonstrated that the Mako enhanced workflow improved the limb length correction and femoral offset when compared to conventional THA. As far as we are aware, there are no previous published studies comparing the outcomes following femoral preparation of the enhanced with express workflow, which is crucial to further advance our knowledge in this emerging field.^15^ Thus, the primary objective of this study was to assess the accuracy of each workflow in estimating hip length discrepancy and combined offset, following their application for femoral canal preparation when performing a Mako robotic arm-assisted THA. Secondary outcomes of interest included comparison of the measured combined offset and hip length discrepancy, surgical time, and incidence of postoperative instability/dislocation, as well as pin-site related complications between the two groups.

## Methods

**Fig. 1:**
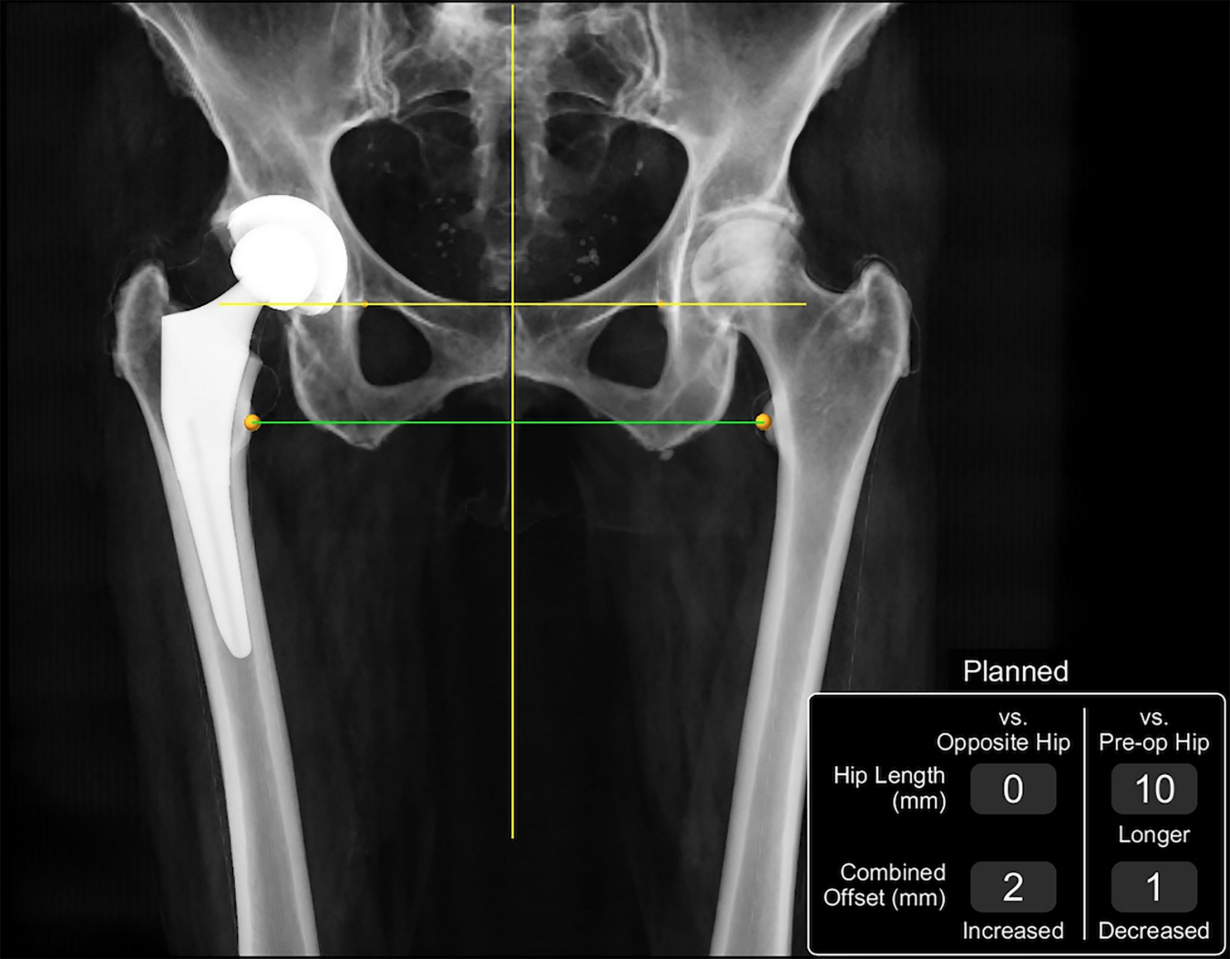
Example of prosthesis templating, with expected correction in hip length and combined offset displayed using the Mako system.

This is a retrospective cohort study reported in accordance with the Strengthening the Reporting of Observational Studies in Epidemiology (STROBE) guidelines.^16^ Human research ethics committee approval was sought and obtained prior to the commencement of this study (approval no. HREC/2024/MNHB/112604). Consecutive patients who underwent an elective unilateral Mako robotic arm-assisted THA via the posterior approach at our institution between January 2019 and July 2024 were included in the study. All included patients received a combination of Accolade II and Trident implants (Stryker), under the supervision of a fellowship-trained consultant orthopaedic surgeon (AMS, WJD), who routinely recorded their final measurments on the Mako system. We excluded those who received cemented implants,^1^ as the combined offset and hip length correction may be altered during the cementing process;^2^ those where the final combined offset and hip length discrepancy were not recorded in the Mako system or where poor-quality postoperative imaging precluded accurate estimation of these measures;^3^ those with contralateral or bilateral simultaneous THA;^4^ those with previous contralateral hip surgery or significant acetabular deformity (Paprosky type 2A and above); and those performed using the anterior approach.^5^ Preoperative CT scan was obtained, and templating was performed using the Mako software to ensure optimal cup and stem placement (Figure 1). The decision to proceed with either enhanced or express workflow was left to the discretion of the operating surgeon. In this study, all surgeons were proficient in, and routinely used both workflows, depending on the surgical goals. They have all surpassed the learning curve for robotic arm-assisted THA. Postoperatively, all patients were allowed to fully weightbear with no hip precautions. Only a single perioperative dose of intravenous antibiotics was administered prior to skin incision. Routine postoperative roentgenograms were obtained for all patients, who were followed up for a minimum of 12 months. The Mako software used in this study was version 3.5.

### Patient characteristics

A total of 81 patients were identified using our inclusion and exclusion criteria. Of these patients, 61 received femoral sided preparation using the enhanced workflow, while 20 received femoral sided preparation using the express workflow. There was no significant difference between the two groups in terms of baseline demographic characteristics, underlying pathology, degree of osteoarthritis, or severity of acetabular defects (Table I).

**Table I.** Baseline demographic information and surgical factors.

Variable	Enhanced workflow (n = 61)	Express workflow (n = 20)	p-value*
Mean age at surgery, yrs (range)	56 (47 to 63)	54 (46 to 59)	0.428
**Sex, n (%)**			0.448
Male	30 (49.18)	12 (60.00)	
Female	31 (50.82)	8 (40.00)	
Mean BMI, kg/m^2^ (range)	29.33 (25.97 to 33.16)	29.75 (26.88 to 37.31)	0.256
**ASA grade, n (%)**			0.669
1	8 (13.11)	2 (10.00)	
2	39 (63.93)	11 (55.00)	
3	13 (21.31)	7 (35.00)	
4	1 (1.64)	0 (0)	
**Comorbidities, n (%)**			
Hypertension	22 (36.07)	4 (20.00)	0.270
Cardiac arrhythmia/ischaemic heart disease	1 (1.64)	0 (0)	1.000
Cardiac failure	1 (1.64)	0 (0)	1.000
Severe respiratory disease	3 (4.92)	0 (0)	0.571
Chronic liver disease	1 (1.64)	1 (5.00)	0.435
Chronic kidney disease	3 (4.92)	0 (0)	0.571
Cerebrovascular disease	3 (4.92)	0 (0)	0.571
Diabetes	5 (8.20)	4 (20.00)	0.475
Seizure disorder	0 (0)	0 (0)	1.000
Smoker	14 (22.95)	1 (5.00)	0.109
**Underlying diagnosis, n (%)**			0.646
Osteoarthritis	57 (93.44)	18 (90.00)	
Developmental hip dysplasia	2 (3.28)	1 (5.00)	
Perthes’ disease	1 (1.64)	0 (0)	
Slipped capital femoral epiphysis	0 (0)	1 (5.00)	
Post-traumatic arthritis	1 (1.64)	0 (0)	
**Paprosky’s classification, n (%)** †			0.136
1	10 (16.39)	4 (20.00)	
2A	5 (8.20)	5 (25.00)	
2B	35 (57.38)	7 (35.00)	
2C	5 (8.20)	1 (5.00)	
3A	6 (9.84)	2 (10.00)	
3B	0 (0)	1 (5.00)	
**Tönnis classification, n (%)** ‡			0.125
1	9 (14.75)	4 (20.00)	
2	20 (32.79)	2 (10.00)	
3	32 (52.46)	14 (70.00)	

* Continuous variables were displayed as median (IQR) and compared using the Mann-Whitney U test, whereas discrete variables were displayed as count (percentage) and compared using Fisher’s exact test.

† Classification of acetabular defects.

‡ Classification of hip osteoarthritis.

ASA, American Society of Anesthesiologists.

### Enhanced workflow

The enhanced workflow involves acetabular and femoral registration. After adequate positioning and skin preparation, three bone pins were inserted over the lateral aspect of the iliac crest via stab incisions for attachment of the pelvic array. Following this, two bone pins were inserted in the distal femur for attachment of the femoral array. The approach was then made, and checkpoints were placed on the lateral aspect of the greater trochanter (femoral checkpoint) and upper edge of the acetabulum (pelvic checkpoint). Safe surgical dislocation of the hip was performed, and the femur was registered onto the robotic system. Femoral neck osteotomy and sequential femoral broaching was then performed under robotic guidance. The planned femoral component anteversion, hip length correction, and combined offset can be checked throughout this process. After that, we proceeded to register the acetabulum. The acetabular preparation, trial, and definitive component insertion is performed by the robotic arm to facilitate optimal acetabular component positioning. Finally, the femoral component is inserted, and the hip length correction and combined offset is checked and recorded.

### Express workflow

The express workflow involves only acetabular registration. An electrocardiogram tab was placed on the centre of the patella and secured with Tegaderm dressing prior to skin preparation. A stockinette was then used in the draping process, secured with sterile crepe, while ensuring that the tab remained palpable. Pelvic pins, as well as pelvic and femoral checkpoints, were placed using the technique described in the enhanced workflow. Safe surgical dislocation and femoral neck osteotomy were then performed. The remaining steps of acetabular registration, preparation, trial, and definitive component insertion were performed similarly to that of the enhanced workflow. Following this, the femur was broached, trialled, and definitive components were inserted freehand by the surgeon. The surgeon was able to measure the change in leg length and combined offset at any time during the procedure with either the trial or definitive components in situ by reducing the hip and registering with the pointer the palpable tab on the patellar tendon (for limb position) and divot on the femoral checkpoint from which the software calculates leg length and combined offset values.

### Statistical analysis

Basic demographic data, underlying diagnosis, degree of hip degeneration classified using the Tönnis classification,^17^ and degree of acetabular defect using the Paprosky classification^18^ were collected. None of our included patients had femoral-sided defects. The primary outcome of interest was to assess the accuracy of each workflow in estimating hip length discrepancy and combined offset. We compared the registered final combined offset and hip length discrepancy with preoperative and immediate postoperative radiographs, using the following indices: hip length discrepancy as compared to the contralateral side and combined offset as compared with the contralateral side. For the measurement of hip length discrepancy and combined offset, postoperative roentgenograms were first calibrated using the femoral head as a guide. Subsequent measurements were then made based on the technique described by Laggner et al.^19^ Secondary outcomes of interest included comparison of the measured combined offset and hip length discrepancy, surgical time, incidence of postoperative instability/dislocation, as well as pin-site related complications between the two groups. All measurements were performed by a speciality registrar (VP) and verified by a fellowship-trained consultant orthopaedic surgeon (AS).

**Fig. 2:**
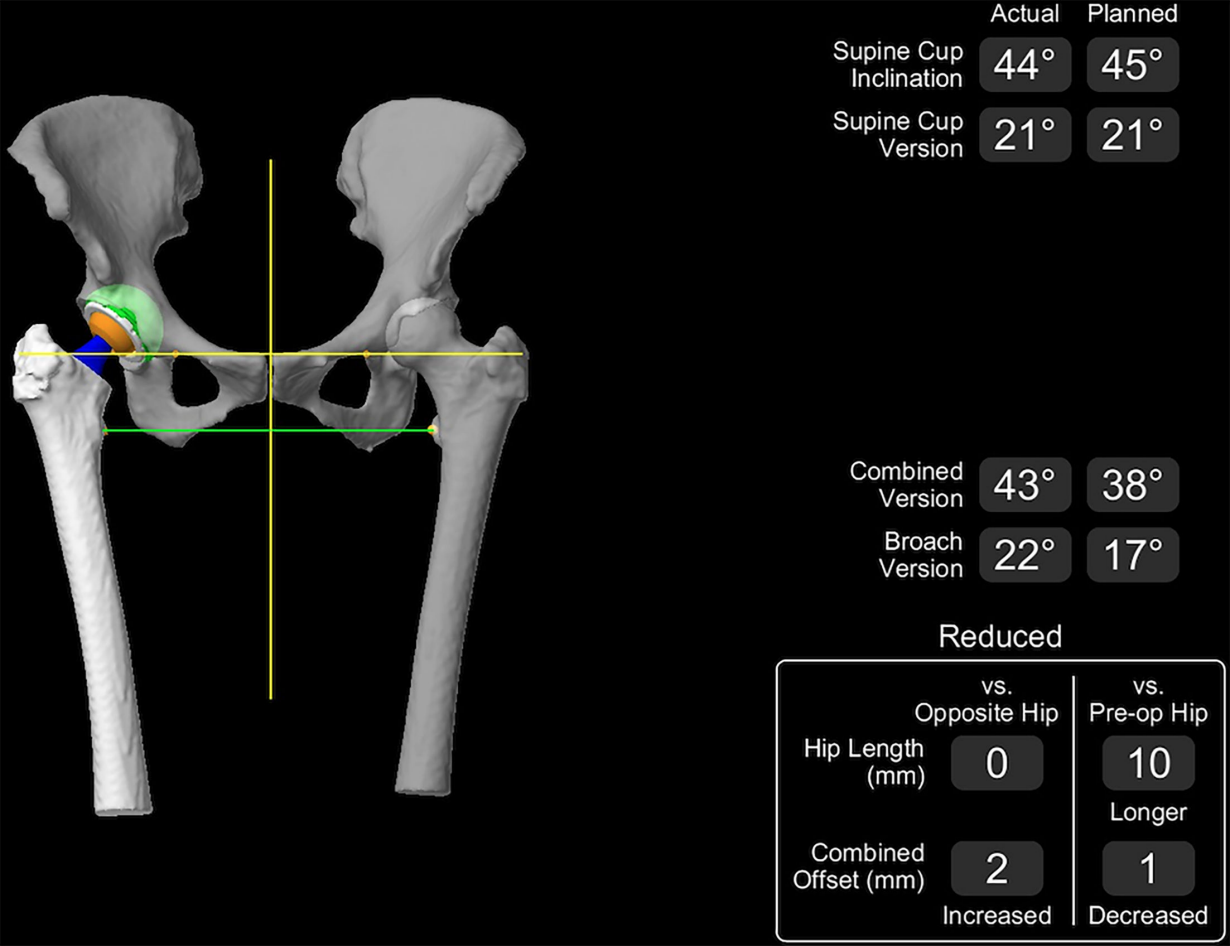
Example of Mako derived values for hip length discrepancy and combined offset following reduction of prosthesis.

The agreement between the registered final combined offset and hip length discrepancy on the Mako robotic system (Figure 2) as compared with preoperative and postoperative radiographs were illustrated using Bland-Altman’s plot and assessed visually. Theil’s median slope, a generalization of the Hodges-Lehmann median difference to the case of a non-binary variable, is used to quantify the magnitude of this difference. The robust 95% CIs of the median difference was then computed using the technique described by Newson.^20-22^ Continuous variables were displayed as median (IQR) and compared using the Mann-Whitney U test, whereas discrete variables were displayed as count (percentage) and compared using Fisher’s exact test. All analyses were performed using Stata v. 18.0 (StataCorp, USA). The threshold for statistical significance was set at p < 0.05.

## Results

### Primary outcomes

**Fig. 3:**
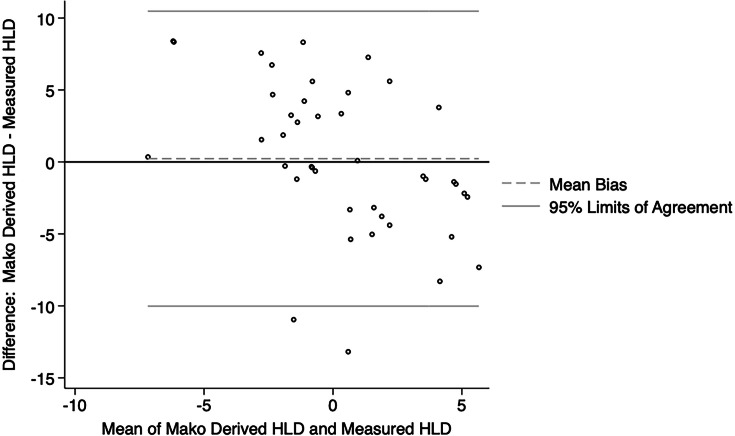
Bland-Altman plot showing agreement between Mako derived hip length discrepancy (HLD) and HLD measured in postoperative imaging for the enhanced workflow group.

**Fig. 4:**
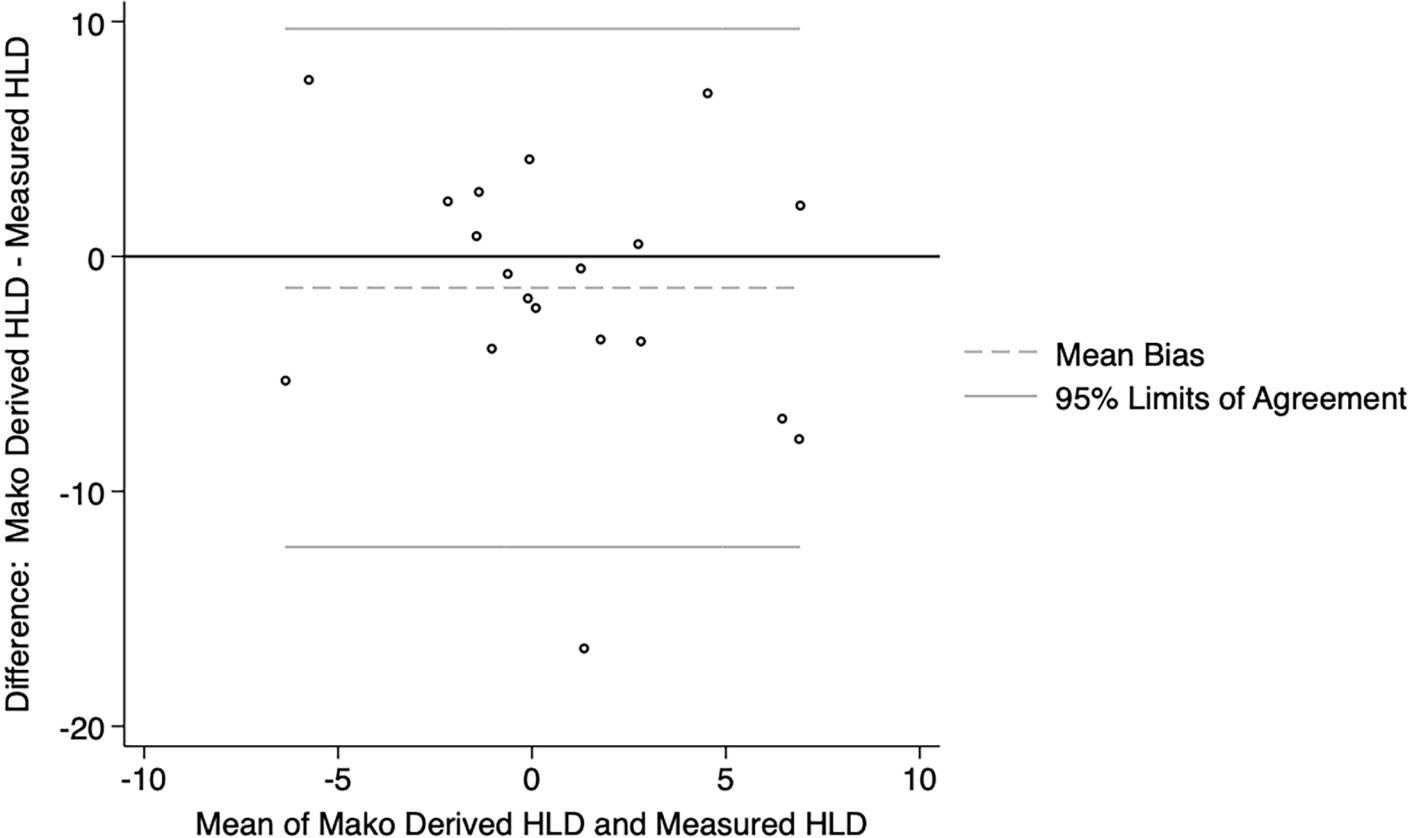
Bland-Altman plot showing agreement between Mako derived hip length discrepancy (HLD) and HLD measured in postoperative imaging for the express workflow group.

The Bland-Altman plots showed reasonable agreement between the Mako derived hip length discrepancy values and those measured on postoperative imaging for both the enhanced (Figure 3) and express (Figure 4) workflow. The Mako derived hip length discrepancy values were not significantly different from that measured on postoperative imaging for both the enhanced (median 0.30 mm; 95% CI -3.18 to 1.37) and express workflow (median 0.73 mm; 95% CI -2.18 to 3.60).

**Fig. 5:**
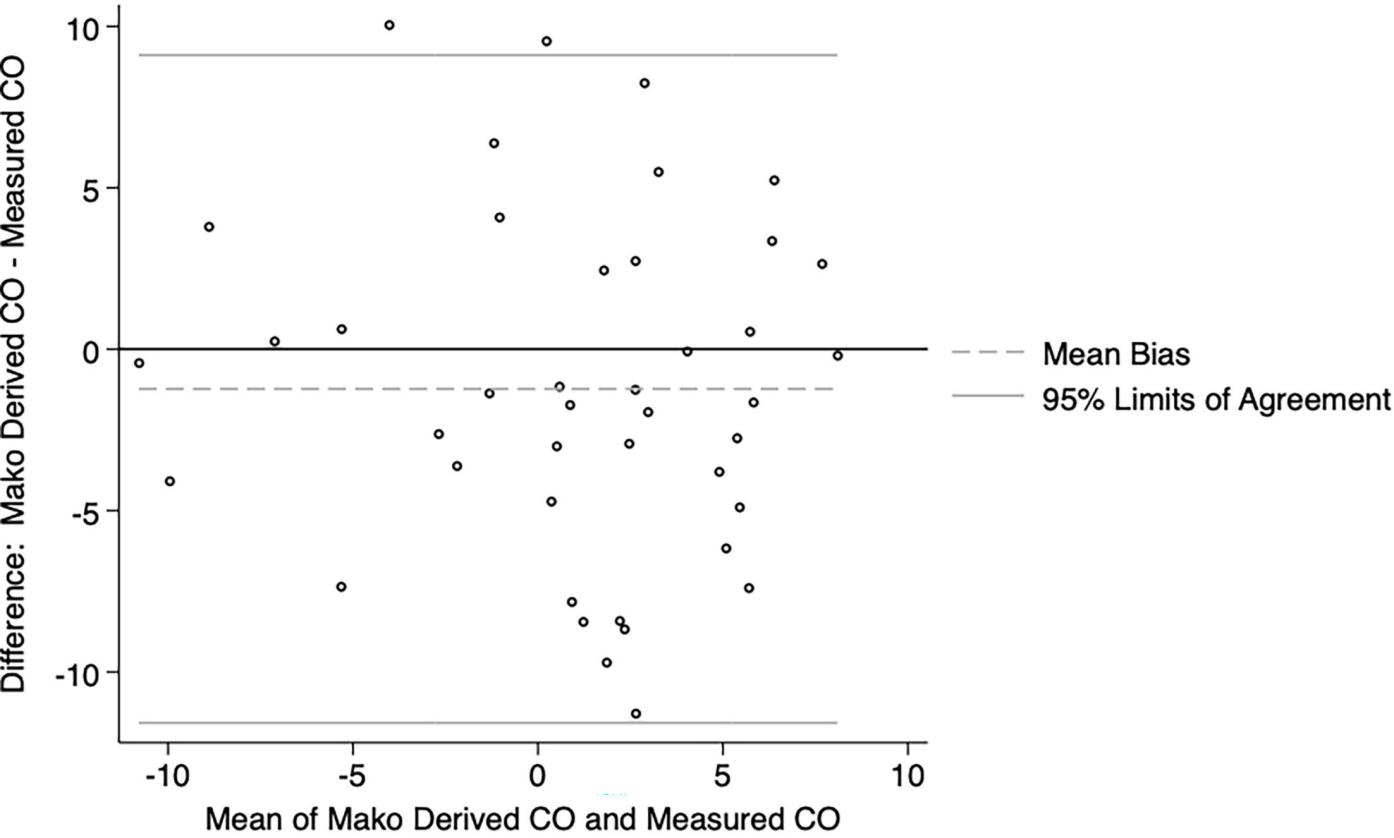
Bland-Altman plot showing agreement between Mako derived combined offset (CO) and CO measured in postoperative imaging, as compared to the contralateral side, for the enhanced workflow group.

**Fig. 6:**
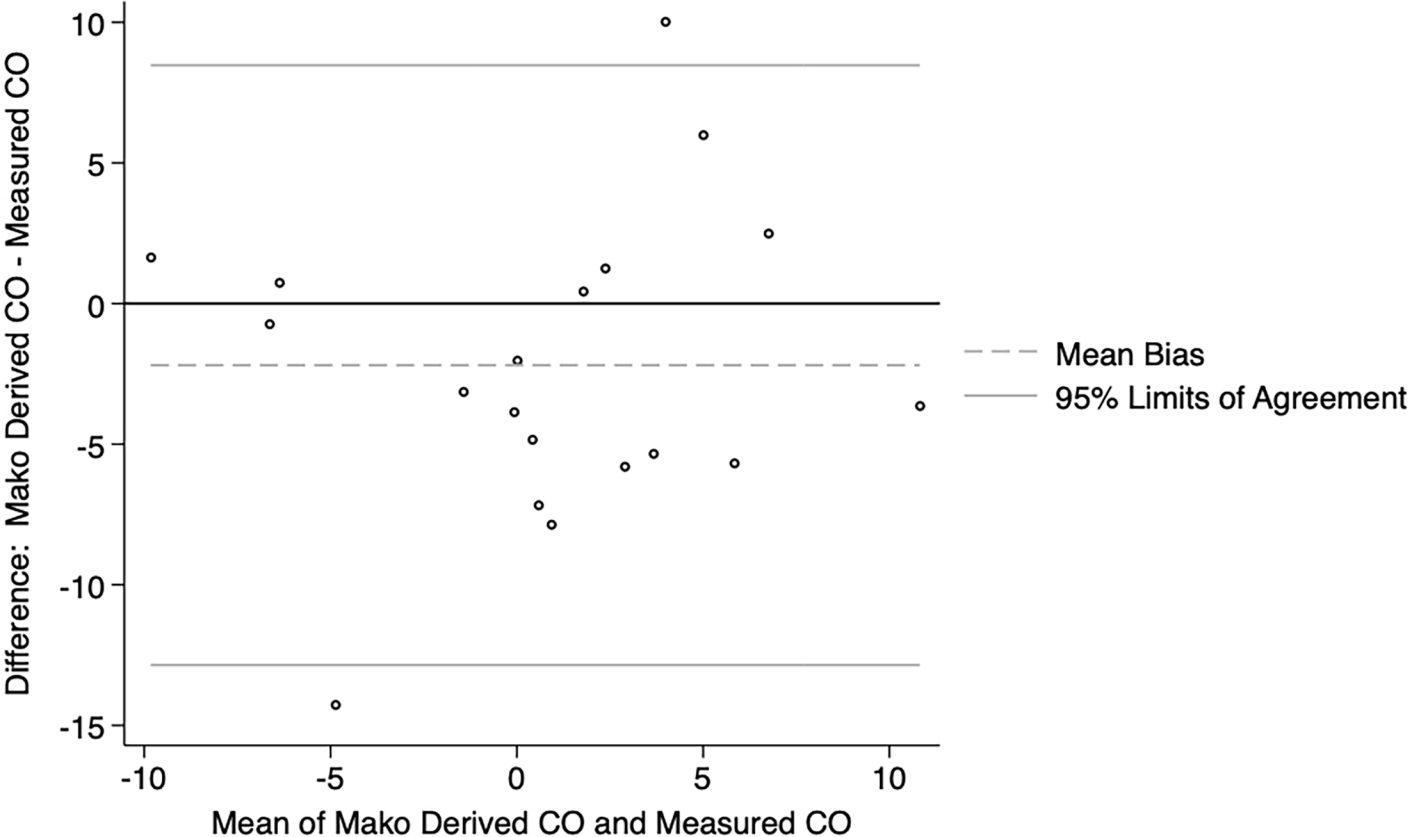
Bland-Altman plot showing agreement between Mako derived combined offset (CO) and CO measured in postoperative imaging, as compared to the contralateral side, for the express workflow group.

Similarly, for the measurement of combined offset compared to that on the contralateral side, the Bland-Altman plots also showed reasonable agreement between Mako derived values and those measured on radiographs for both the enhanced (Figure 5) and express (Figure 6) workflows. The Mako derived combined offset difference with the contralateral side were not significantly different to that measured on postoperative imaging for both the enhanced (median 1.50 mm; 95% CI -0.25 to 3.00) and the express workflows (median 3.13 mm; 95% CI -0.75 to 5.33). These findings are summarized in Table II.

**Table II.** Result summary of primary outcomes.

	Enhanced workflow		Express workflow	
Outcome	Median	95% CI	Median	95% CI
Difference between Mako derived and actual hip length discrepancy, mm	-0.30	-3.18 to 1.37	0.73	-2.18 to 3.60
Difference between Mako derived and actual combined offset as compared to contralateral values, mm	1.50	-0.25 to 3.00	3.13	-0.75 to 5.33

### Secondary outcomes

There were no significant differences in the measured hip length discrepancy (p = 0.254), combined offset difference when compared to the contralateral values (p = 0.425), or in surgical time (p = 0.548) between the two workflows. Additionally, there were no dislocations/instability or pin-site related complications between the two cohorts. There was one complication of note. This patient, in the express group, sustained an intraoperative acetabular fracture during cup impaction necessitating reduction in weightbearing status postoperatively. Our secondary outcomes are summarized in Table III.

**Table III.** Result summary of secondary outcomes.

Outcome	Enhanced workflow	Express workflow	p-value*
Median measured hip length discrepancy, mm (IQR)	-0.62 (-3.22 to 3.98)	1.11 (-2.02 to 5.21)	0.254
Median measured difference in combined offset as compared with contralateral value, mm (IQR)	3.53 (-0.39 to 6.67)	1.93 (0.58 to 5.18)	0.425
Median surgical time, mins (IQR)	99 (92.00 to 116.00)	101.50 (94.00 to 118.00)	0.548
Instability/dislocation in the follow-up period, n (%)	0/74 (0)	0/26 (0)	1.000
Pin-site related complications in the follow-up period, n (%)	0/74 (0)	0/26 (0)	1.000

* Continuous variables were displayed as median (IQR) and compared using the Mann-Whitney U test, whereas discrete variables were displayed as count (percentage) and compared using Fisher’s exact test.

## Discussion

Restoration of native combined offset and avoiding limb length discrepancies are crucial technical objectives of the THA. The results of our analysis demonstrated that the Mako robotic arm-assisted THA had reasonable agreement with measured values of hip length discrepancy and combined offset. A substantial proportion of our included patients had a degree of acetabular defects, with the most common being Paprosky type 2B. Both workflows accurately estimated hip length discrepancies and combined offset when compared to contralateral values in patients with single-sided THA. Crucially, none of our included patients had a pin-site related complication or prosthetic hip dislocation/instability.

**Fig. 7:**
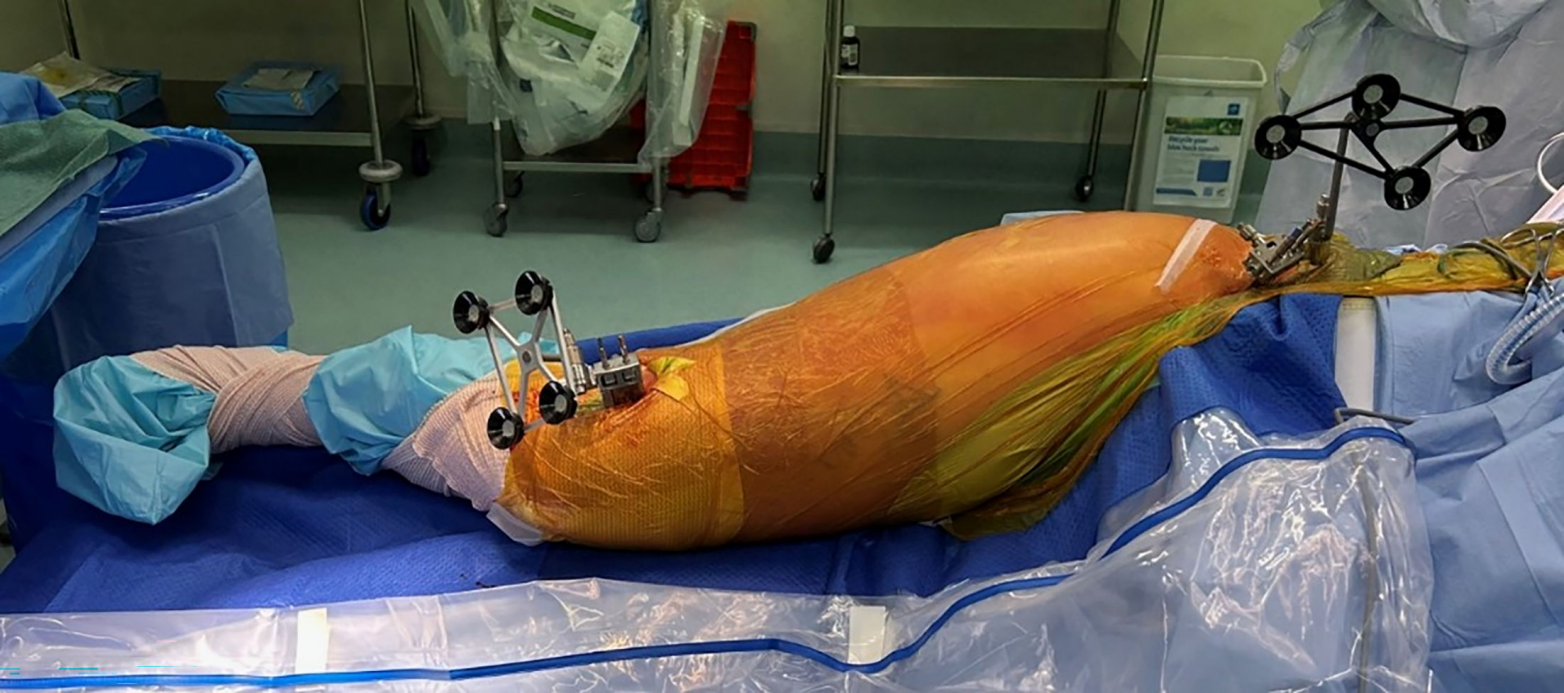
Intraoperative photograph demonstrating distal pin placement technique.

The standard position for insertion for the femoral tracker is via a pin into the greater trochanteric region of the proximal femur.^12^ Due to the location of this tracker, a number of complications had been experienced by our surgeons, including limited tracker visibility, the requirement for a larger skin incision, and loosening of the tracker fixation due to poor cancellous bone quality. As such, an alternative technique of pin placement has been adopted at our institution over the past few years as it is thought to be less invasive and to have fewer complications. For the execution of this technique, the femoral pins are inserted more distally on the femur, that is, in the lateral epicondylar region of the knee. We advocate for this to be strongly considered by robotic surgeons using the Mako system, particularly in the case of severe osteoporosis. Additionally, with the expansion of the Mako robotic system capabilities to include revision arthroplasty, this may also represent an exciting avenue for further development. In our series, the described technique demonstrated reliability comparable to that of the express workflow. We have illustrated our technique in Figure 7.

According to the most recent Australian Orthopaedic Association National Joint Replacement Registry report,^23^ revision for dislocation, limb length discrepancy, and implant malposition cumulatively accounted for almost a quarter of revisions. In our series, although both the enhanced and express workflows accurately estimated the combined offset when compared to the contralateral hip, the enhanced workflow trended toward greater accuracy (median 1.50 mm vs 3.13 mm). An increase in femoral offset can lead to trochanteric pain due to irritation of the soft tissues and acceleration of polyethylene liner wear, while a decrease in femoral offset can lead to impingement and instability.^24^ Additionally, several studies have shown that limb length discrepancies greater than 5 mm to 10 mm may be perceived by patients, particularly if the operated leg is lengthened, which can negatively affect patient-reported outcome measures and function.^25-27^ Both the enhanced and express protocols reliably allowed for restoration of hip length when compared to the contralateral side. The number of outliers (greater than 95% limits of agreement) for estimation of hip length discrepancy was 2/61 (3.28%) and 1/20 (5.00%), whereas the number of outliers for estimation of combined offset was 2/61 (3.28%) and 2/20 (10.00%).

Given the projected rise in robotic arm-assisted THA,^28,29^ this research is timely to better inform surgeons on the anticipated benefits and potential risks in adopting either workflow options in clinical practice. Nevertheless, our study has several limitations that need to be addressed. First, this study did not include a control group with patients undergoing conventional THA. Thus, we are unable to assess whether robotic arm-assisted THA was superior to conventional THA in restoring native hip joint biomechanics. Second, the postoperative measurements for combined offset and hip length discrepancy were measured using calibrated roentgenograms. Ideally, CT-based measurements alongside assessment of acetabular component positioning and combined anteversion are required to provide a more holistic and accurate overview of this subject.^30^ Third, we were unable to comment whether there is a subset of patient with specific pattern of acetabular defects or underlying pathology who may benefit from one workflow over another. Fourth, we only included patients who had a normal native hip on the contralateral side, limiting the generalizability of this study. The rationale for this is to prevent skewed results due to contralateral hip pathology or unsatisfactory prosthetic hip arthroplasty. Furthermore, our small and unequal sample size is also a major limitation. To provide a genuine comparison between the two groups, we have only included surgeons who routinely used both workflows and recorded their final implant position on the Mako system. Additionally, a useful metric for future studies is to compare planned with achieved femoral length and offset. In this study, we were unable to collect information on preoperative planning. Lastly, our results are only applicable to users of the Mako robotic system who received uncemented implants via a posterior approach, further limiting its generalizability.

In conclusion, both the enhanced and express workflows when performing a Mako robotic arm-assisted THA provided excellent clinical results with minimal risk of complications. Good agreement was visualized on the Bland-Altman plots between the Mako derived measurements of hip length discrepancies and combined offset, with very few outliers. Both workflows accurately estimated hip length and combined offset, although the enhanced workflow may provide a better estimation of combined offset. Surgeons should therefore choose the technique which best adapts to their practice. Further research incorporating other approaches and robotic designs are required to improve the generalizability of these findings.

**Take home message**

- Both the express and enhanced workflow for the Mako robotic-assisted total hip arthroplasty system provided accurate measurements of hip length and combined offset with no difference in surgical time or complication rate.

## Data Availability

The data that support the findings for this study are available to other researchers from the corresponding author upon reasonable request, subject to ethical approval and institutional requirements.

## References

[1] Learmonth ID Young C Rorabeck C The operation of the century: total hip replacement Lancet 2007 370 9597 1508 1519 10.1016/S0140-6736(07)60457-7 17964352

[2] Pabinger C Lothaller H Portner N Geissler A Projections of hip arthroplasty in OECD countries up to 2050 Hip Int 2018 28 5 498 506 10.1177/1120700018757940 29783896

[3] Matharu GS Culliford DJ Blom AW Judge A Projections for primary hip and knee replacement surgery up to the year 2060: an analysis based on data from The National Joint Registry for England, Wales, Northern Ireland and the Isle of Man Ann R Coll Surg Engl 2022 104 6 443 448 10.1308/rcsann.2021.0206 34939832 PMC9157920

[4] Johnston RC Brand RA Crowninshield RD Reconstruction of the hip. A mathematical approach to determine optimum geometric relationships J Bone Joint Surg Am 1979 61 5 639 652 457709

[5] Bullock EKC Brown MJ Clark G Plant JGA Blakeney WG Robotics in total hip arthroplasty: current concepts J Clin Med 2022 11 22 6674 10.3390/jcm11226674 36431151 PMC9695933

[6] St Mart J-P Goh EL Shah Z Robotics in total hip arthroplasty: a review of the evolution, application and evidence base EFORT Open Rev 2020 5 12 866 873 10.1302/2058-5241.5.200037 33425375 PMC7784137

[7] Kamara E Robinson J Bas MA Rodriguez JA Hepinstall MS Adoption of robotic vs fluoroscopic guidance in total hip arthroplasty: is acetabular positioning improved in the learning curve? J Arthroplasty 2017 32 1 125 130 10.1016/j.arth.2016.06.039 27499519

[8] Kong X Yang M Jerabek S Zhang G Chen J Chai W A retrospective study comparing a single surgeon’s experience on manual versus robot-assisted total hip arthroplasty after the learning curve of the latter procedure - A cohort study Int J Surg 2020 77 174 180 10.1016/j.ijsu.2020.03.067 32259592

[9] Domb BG El Bitar YF Sadik AY Stake CE Botser IB Comparison of robotic-assisted and conventional acetabular cup placement in THA: a matched-pair controlled study Clin Orthop Relat Res 2014 472 1 329 336 10.1007/s11999-013-3253-7 23990446 PMC3889439

[10] O’Connor PB Thompson MT Esposito CI et al. The impact of functional combined anteversion on hip range of motion: a new optimal zone to reduce risk of impingement in total hip arthroplasty Bone Jt Open 2021 2 10 834 841 10.1302/2633-1462.210.BJO-2021-0117.R1 34633223 PMC8558443

[11] Sugano N Maeda Y Fuji H et al. Accuracy of femoral component anteversion in robotic total hip arthroplasty Bone Joint J 2024 106-B 3 Supple A 104 109 10.1302/0301-620X.106B3.BJJ-2023-0840.R1 38425294

[12] MAKO THA Surgical Guide Stryker 2015 https://www.strykermeded.com/media/2042/mako-tha-surgical-technique.pdf date last accessed 28 January 2026

[13] Kayani B Konan S Thakrar RR Huq SS Haddad FS Assuring the long-term total joint arthroplasty Bone Joint J 2019 101-B 1_Supple_A 11 18 10.1302/0301-620X.101B1.BJJ-2018-0377.R1 30648491

[14] Nawabi DH Conditt MA Ranawat AS et al. Haptically guided robotic technology in total hip arthroplasty: a cadaveric investigation Proc Inst Mech Eng H 2013 227 3 302 309 10.1177/0954411912468540 23662346

[15] Khatri C Metcalfe A Wall P Underwood M Haddad FS Davis ET Robotic trials in arthroplasty surgery Bone Joint J 2024 106-B 2 114 120 10.1302/0301-620X.106B2.BJJ-2023-0711.R1 38295854

[16] von Elm E Altman DG Egger M et al. The strengthening the reporting of observational studies in epidemiology (STROBE) statement: guidelines for reporting observational studies Ann Intern Med 2007 147 8 573 577 10.7326/0003-4819-147-8-200710160-00010 17938396

[17] Busse J Gasteiger W Tönnis D Eine neue Methode zur röntgenologischen Beurteilung eines Hüftgelenkes — Der Hüftwert Arch orthop Unfall-Chir 1972 72 1 1 9 10.1007/BF00415854 5020681

[18] Telleria JJM Gee AO Classifications in brief: Paprosky classification of acetabular bone loss Clin Orthop Relat Res 2013 471 11 3725 3730 10.1007/s11999-013-3264-4 23996098 PMC3792247

[19] Laggner R Oktarina A Windhager R Bostrom MPG Changes in leg length and hip offset in navigated imageless vs. conventional total hip arthroplasty Sci Rep 2023 13 1 17161 10.1038/s41598-023-44009-6 37821499 PMC10567748

[20] Newson R Confidence intervals for rank statistics: percentile slopes, differences, and ratios Stata J 2006 6 4 497 520 10.1177/1536867X0600600404

[21] Newson R Parameters behind “Nonparametric” Statistics: Kendall’s tau, Somers’ D and median differences Stata J 2002 2 1 45 64 10.1177/1536867X0200200103

[22] Theil H A rank-invariant method of linear and polynomial regression analysis Indagationes mathematicae 1950 12 85 173

[23] Lewis PG McAuliffe MJ Stoney JD Knee and Shoulder Arthroplasty: 2025 annual report Adelaide, South Australia Australian Orthopaedic Association National Joint Replacement Registry 2025 https://aoanjrr.sahmri.com/annual-reports-2025 date last accessed 28 January 2026

[24] Shapira J Chen SL Rosinsky PJ et al. The effect of postoperative femoral offset on outcomes after hip arthroplasty: a systematic review J Orthop 2020 22 5 11 10.1016/j.jor.2020.03.034 32273666 PMC7132120

[25] Zhang Y He W Cheng T Zhang X Total hip arthroplasty: leg length discrepancy affects functional outcomes and patient’s gait Cell Biochem Biophys 2015 72 1 215 219 10.1007/s12013-014-0440-4 25516289

[26] Konyves A Bannister GC The importance of leg length discrepancy after total hip arthroplasty J Bone Joint Surg Br 2005 87-B 2 155 157 10.1302/0301-620x.87b2.14878 15736733

[27] Keršič M Dolinar D Antolič V Mavčič B The impact of leg length discrepancy on clinical outcome of total hip arthroplasty: comparison of four measurement methods J Arthroplasty 2014 29 1 137 141 10.1016/j.arth.2013.04.004 23680505

[28] Lewis P Gill D McAuliffe M McDougall C Stoney J Vertullo C et al. Hip, Knee and Shoulder Arthroplasty: 2024 Annual Report South Australia Australian Orthopaedic Association National Joint Replacement Registry 2024

[29] The Eleventh Annual Report of the AJRR on Hip and Knee Arthroplasty American Academy of Orthopaedic Surgeons: American Joint Replacement Registry 2024 https://connect.registryapps.net/hubfs/AJRR/AJRR%202024%20Annual%20Report.pdf?utm_medium=email&utm_source=transaction date last accessed 28 January 2026

[30] Hardwick-Morris M Wigmore E Twiggs J Miles B Jones CW Yates PJ Leg length discrepancy assessment in total hip arthroplasty: is a pelvic radiograph sufficient? Bone Jt Open 2022 3 12 960 968 10.1302/2633-1462.312.BJO-2022-0146.R1 36510730 PMC9783271

